# Relationship Between Chronic Stress and Heart Rate Over Time Modulated by Gender in a Cohort of Office Workers: Cross-Sectional Study Using Wearable Technologies

**DOI:** 10.2196/18253

**Published:** 2020-09-09

**Authors:** Alex Wilhelmus Jacobus van Kraaij, Giuseppina Schiavone, Erika Lutin, Stephan Claes, Chris Van Hoof

**Affiliations:** 1 Holst Centre imec-the Netherlands Eindhoven Netherlands; 2 OnePlanet Research Center imec-the Netherlands Wageningen Netherlands; 3 Faculty of Natural Sciences, Math and Informatics (FNWI) Radboud University Nijmegen Netherlands; 4 Electrical Engineering-ESAT KU Leuven Leuven Belgium; 5 imec-Belgium Heverlee Belgium; 6 University Psychiatric Center & Department of Neurosciences KU Leuven Leuven Belgium

**Keywords:** chronic stress, heart rate, circadian rhythm, gender, age, wearable device

## Abstract

**Background:**

Chronic stress is increasing in prevalence and is associated with several physical and mental disorders. Although it is proven that acute stress changes physiology, much less is known about the relationship between physiology and long-term stress. Continuous measurement of vital signs in daily life and chronic stress detection algorithms could serve this purpose. For this, it is paramount to model the effects of chronic stress on human physiology and include other cofounders, such as demographics, enabling the enrichment of a population-wide approach with individual variations.

**Objective:**

The main objectives of this study were to investigate the effect of chronic stress on heart rate (HR) over time while correcting for weekdays versus weekends and to test a possible modulation effect by gender and age in a healthy cohort.

**Methods:**

Throughout 2016 and 2017, healthy employees of technology companies were asked to participate in a 5-day observation stress study. They were required to wear two wearables, of which one included an electrocardiogram sensor. The derived HR was averaged per hour and served as an output for a mixed design model including a trigonometric fit over time with four harmonics (periods of 24, 12, 8, and 6 hours), gender, age, whether it was a workday or weekend day, and a chronic stress score derived from the Perceived Stress Scale (PSS) as predictors.

**Results:**

The study included 328 subjects, of which 142 were female and 186 were male participants, with a mean age of 38.9 (SD 10.2) years and a mean PSS score of 13.7 (SD 6.0). As main effects, gender (χ^2^_1_=24.02, *P*<.001); the hour of the day (χ^2^_1_=73.22, *P*<.001); the circadian harmonic (χ^2^_2_=284.4, *P*<.001); and the harmonic over 12 hours (χ^2^_2_=242.1, *P*<.001), over 8 hours (χ^2^_2_=23.78, *P*<.001), and over 6 hours (χ^2^_2_=82.96, *P*<.001) had a significant effect on HR. Two three-way interaction effects were found. The interaction of age, whether it was a workday or weekend day, and the circadian harmonic over time were significantly correlated with HR (χ^2^_2_=7.13, *P*=.03), as well as the interaction of gender, PSS score, and the circadian harmonic over time (χ^2^_2_=7.59, *P*=.02).

**Conclusions:**

The results show a relationship between HR and the three-way interaction of chronic stress, gender, and the circadian harmonic. The modulation by gender might be related to evolution-based energy utilization strategies, as suggested in related literature studies. More research, including daily cortisol assessment, longer recordings, and a wider population, should be performed to confirm this interpretation. This would enable the development of more complete and personalized models of chronic stress.

## Introduction

### Background

The number of individuals having chronic stress and stress-related mental disorders, such as depression, is increasing globally [1]. While in most cases, the stress response of the human body protects the body in harmful environments, when stress occurs for a prolonged period, it can have several negative health effects [2]. As a matter of fact, chronic stress is known to increase the risk of developing a range of mental and physical disorders [3]. Examples of mental disorders are burnout, depression, and anxiety disorders, while related physical disorders include gastrointestinal disorders, obesity, diabetes, and heart diseases [3]. Moreover, chronic stress is often referred to as an economical problem. In 2018, a review was published on the costs of illness for work-related stress, suggesting that total costs per country could range from US $221 million to US $187 billion each year [4]. For these reasons, it is of critical importance to successfully monitor and manage chronic stress in the entire population, without losing individual-based focus as in current one-on-one psychotherapy.

As described by Kaplan [5], there are many challenges that researchers as well as doctors and therapists face to assess and monitor chronic stress levels in daily life. Although these challenges are already known for a long time, they have not been overcome. Stress questionnaires are the most common and convenient tools used for assessing chronic stress. Questionnaires, such as the Perceived Stress Scale (PSS) that is designed to assess the long-term stress effect (ie, stress over the last month), have been proven to have adequate reliability across different cohorts [6-8]. Nevertheless, they remain subjective measurements that are subject to recall errors and unable to capture the impact of stress on normal physiological functioning [9]. Over the past years, many algorithms and applications have been developed for acute stress detection based on physiological signals [10]. Predictors used are, for example, heart rate (HR), HR variability, skin conductance, and skin temperature [10-12]. Some algorithms can detect acute stress responses with high accuracy (>90%) in controlled conditions [11,12]. Detection algorithms based on real-time monitoring of physiological parameters could improve the objectivity of stress detection, facilitate capturing early signs of chronic stress, and support stress management in clinical practice, functioning both as awareness and feedback tools for users and therapists.

Most of the efforts in this field have focused, so far, on acute stress detection algorithms, showing the difficulty of generalization and the importance of individual-based models [13,14]. Literature on complete physiological modeling of chronic stress in humans remains poor owing to the complexity and high cost of collecting longitudinal real-world data. This complexity needs to be addressed first by linking long-term physiology measurements to chronic stress. Van Uum et al [15] found elevated hair cortisol levels in patients with severe chronic pain and higher PSS scores. Using a repeated measurement design, Schulz et al [16] reported that the awakening salivary cortisol level should be considered a possible biological correlate of chronic stress, as it was found to be elevated in participants with higher chronic stress levels. Schulz et al also found a gender difference in their study, showing larger increases of morning salivary cortisol levels in women than in men. This gender difference in physiological responses to chronic and acute stress has also been investigated by Jones et al [17]. In this previous study, it was found that chronic stress, measured according to the PSS score, modulates both the cortisol stress response and the cardiovascular stress response differently for male and female individuals. Female individuals had a lower HR at rest for higher PSS scores, whereas among male individuals, this correlation was not found. However, male individuals did show a lower HR while in acute stress for higher PSS scores. This second correlation was not found among female individuals.

When studying effects on physiology over multiple days, the circadian rhythm is used to describe diurnal physiological fluctuations [18]. For example, Cho et al [19] developed a model for mood prediction in patients with mood disorders, using the properties of the cosine curve in HR as one of the predictors. Tsanas et al [20] also stressed the importance of the circadian rhythm in monitoring under free-living conditions. Morelli et al [21] described this circadian rhythm in HR as four harmonics with periods of 24, 12, 8, and 6 hours. Their accurate approximation of the resting HR using these four harmonics makes this modeling suitable for mapping long-term effects on HR. Another important aspect to consider while studying effects on physiology over time is the weekday-weekend difference. In the study by Schlotz et al [22], it was shown that the cortisol response, often related to stress, is different for weekdays versus weekends. Chronic work overload and worrying was found to be related to this rise in cortisol after awakening on workdays, but not on weekend days. This effect was independent of gender. Pantzar et al [23] also found dissimilarities in stress patterns between weekend days and workdays when studying HR in response to stress. This difference was modulated by gender, and there was a smaller effect size by age [23].

By combining the approaches and findings from the studies described above in our study, we analyzed the long-term HR response to chronic stress and its bias for gender and age. Different from previous studies, we used wearable technologies to capture circadian variation of HR over multiple days.

### Objectives of the Study

The main objectives of this study were as follows:

- To investigate the effect of chronic stress on HR over time, while correcting for weekdays versus weekends. For this, chronic stress has been defined as long-term perceived stress.
- To test possible modulation effects by gender and/or age. All interaction effects of these predictors will be included.

The outcomes of this analysis could be integrated into novel computational models of chronic stress detection, according to vital signs collected daily, for example, using widely adopted fitness trackers.

## Methods

### Recruitment

This study is part of the Stress in Work Environment (SWEET) study conducted by imec, which has been described previously [13]. Participants were recruited via email in 11 technology-oriented companies and were all office workers. They were included if they were active employees at the time of the study. No other inclusion or exclusion criteria were applied. Participants did not receive any compensation for participating in the study apart from having a chance at winning a restaurant or travel voucher. Vital signs of the participants were continuously measured for 5 consecutive days, starting on Thursday morning and ending on Monday evening, using two wearables devices (a chest patch and a wristband). All participants provided informed consent before participating in the study.

This observational study was approved by the Research Ethical Committee of UZ Leuven (S57916).

### Data Collection

Before the start of the experiment, participants completed an intake questionnaire. The ﬁrst part inquired about personal information, such as age, gender, health problems, work situation, and lifestyle. Thereafter, four psychological questionnaires were used to assess stress, depression, anxiety, sleep, and general health levels. For this study only perceived chronic stress, measured with the PSS, was considered. The questionnaires were distributed via a dedicated and protected web-based platform. On Thursday morning or afternoon, the participants received and started wearing the two wearable devices, including a chest patch [24] that obtained an electrocardiogram (ECG) at 256 Hz and a Chillband (wristband) that measured skin conductance at 256 Hz and skin temperature at 1 Hz. Both devices also measured three-dimensional accelerometer (ACC) signals at 32 Hz to control for movement artifacts. For this study, only the ECG and ACC data of the wearable positioned on the chest was used and only participants with complete ECG data from Friday 12 AM to Monday 4 PM were included for the analysis. This chest patch has been regulatory approved. Participants were advised to wear the chest patch the entire day and night and were asked to remove the chest patch during vigorous physical activities and to shower with a protective cover, since the chest patch is not waterproof. The battery life of the sensors exceeded the duration of the experiment. Data were recorded and stored on the devices’ internal secure digital cards and uploaded to an internal secure cluster at the end of the experiment. For more details on the entire data collection in the SWEET study, refer to the report by Smets et al [13].

### PSS as a Reference for Chronic Stress

The PSS measures “the degree to which situations in one’s life are appraised as stressful” [25] and represents a measure of the global level of perceived stress over the past month. The PSS was designed for use in community samples of individuals with at least a junior high school education. Three versions of the PSS exist (the subscales PSS-14 items, PSS-10 items, and PSS-4 items). Psychometric properties, namely internal consistency reliability, test-retest reliability, and construct validity, of the PSS have been reviewed across studies by Lee et al in 2012 [26], where it was suggested that the subscale PSS-10 should be used to measure perceived stress both in practice and research. Therefore, in this study, the PSS-10 was used.

### Data Preprocessing

#### Feature Calculation

Based on the ECG data, the HR was derived. Refer to the study by Smets et al [13] for the methods on the quality indicator and peak detection algorithms. The ACC data were used to calculate the magnitude in the x, y, and z directions; the average mean of the x, y, and z coordinates; and the average SD of the x, y, and z coordinates. The absolute differences over time of these three features were summed to retrieve an indicator that was used to identify technical malfunctions. The formulas used to calculate each of the features can be found in Multimedia Appendix 1.

#### Filtering Methods

**Figure 1:**
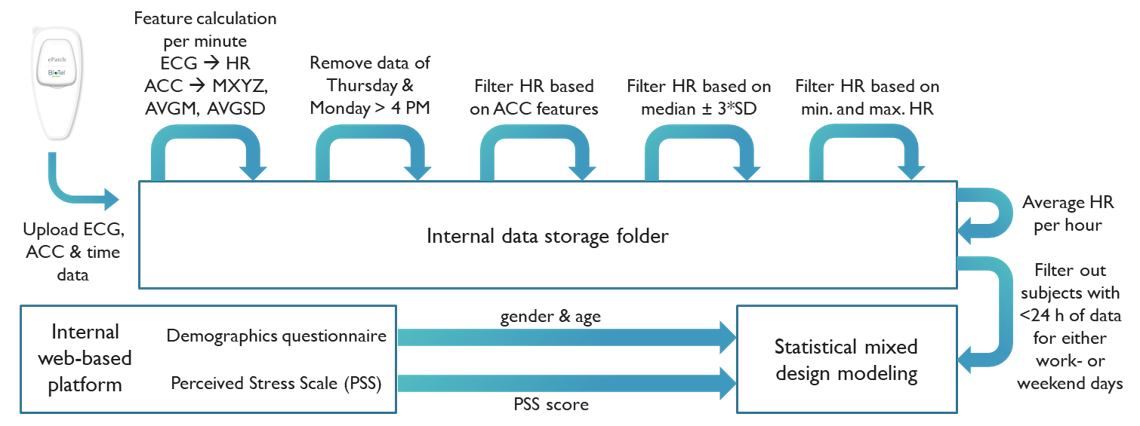
Diagram showing all steps from data upload to model development. ACC: accelerometer; AVGM: average mean acceleration of the x, y, and z coordinates; AVGSD: average standard deviation in acceleration of the x, y, and z coordinates; ECG: electrocardiogram; HR: heart rate; MXYZ: magnitude of acceleration in the x, y, and z directions. See Multimedia Appendix 1 for calculation of the features.

After feature calculation, a data set was obtained with values for the discussed features per minute. First, because all participants started and ended the assessments at different times on Thursday and Monday, the data of Thursday and the data after Monday 4 PM were removed. The data of every participant finally ranged from Friday 12 AM to Monday 4 PM. Second, technical malfunctions (ie, sensor saturation and poor patch adherence) were empirically detected based on the ACC data. For every minute, the indicator retrieved from three ACC features over the 10 closest minutes was calculated. If this value was less than an empirically selected threshold of 0.0005, the patch was probably either removed from the chest during this period or not measuring correctly. These data points were not included in further analysis. To filter out the values affected by artifacts from the remaining HR, two filters were used. First, the individual’s median value ±3 times the SD were used as limits. Second, the minimum HR was set to 30 beats per minute, according to previous studies using HR filters [27,28]. The maximum HR was calculated based on the formula reported by Tanaka et al [29] shown in Multimedia Appendix 1. To assess low-frequency changes in HR, all data were summarized per hour. For the weekday against weekend comparison, Friday and Monday were labeled as no, and Saturday and Sunday were labeled as yes for the variable called weekend. Subjects who did not have 24 or more hours of data for both a weekday and weekend day were excluded from the analysis. Figure 1 shows the data extraction, feature calculation, and filtering methods.

### Statistical Analysis

#### Model Development

The retrieved average HR per hour functioned as the outcome of a mixed design model implemented in RStudio using the “lmer” method from the “lme4” Rpackage. As predictors, the hour of the day, harmonics over time, weekend (yes/no), gender, age, and PSS score were added in the respective order. The subject number was used as a random between-subject intercept and weekend was used as a random within-subject intercept. The time of the day was used in multiple ways based on previous studies on HR over time. First, according to the study by Morelli et al [21], the correlation between HR and time of the day is best described by a four-harmonic fit with periods of 24, 12, 8, and 6 hours. Second, Field et al [30] stated that because of the autoregressive property of physiological signals over time, the “corAR1” function is used as a correlational matrix, which includes the correlations within the hour of the day with random intercepts for each participant and workdays versus weekend days. Therefore, random slopes were used both for the hour of the day as a linear continuous variable and for the four harmonics in time that interacted with the weekend because of the differences in the pattern of HR between workdays and weekend days, as reported previously [31]. The method of comparison was set to “ML” (maximum likelihood), which, according to Field et al [30], is the best way to compare multilevel models. In “control,” the maximum number of iterations was set to 200 and “returnObject” was set to true to make sure the models are not removed because of optimization issues. Not applicable (NA) values were excluded.

Gender, age, and the PSS score were added as fixed effects separately. All two-way interactions and three-way interactions were included. Interaction effects were included either if they were significant or, for the two-way interaction effects, if the three-way interaction effects with both the main effects were significant. Three-way interactions were only included when all related two-way interactions were included as well. Since the study focused on long-term effects, for the interactions with between-subject variables and time, only interactions with the circadian harmonic (the harmonic over 24 hours) were included. For the interactions between weekend and time, all harmonics were included because the random slopes differed for workdays and weekend days.

#### Model Comparison

Initially, the median and interquartile range (IQR) were calculated for the age, PSS score and HR. The HR, age, and PSS score were compared between male and female participants using a Mann-Whitney-Wilcoxon test, while HR on workdays and weekend days was compared using a Wilcoxon signed-rank test. In the final model, we included only interactions that significantly improved the model, which were related to a drop in the Akaike information criterion (AIC) value and an increase in the log likelihood (LogLik) value. The final model was compared to a similar model without the main PSS effect and interaction effects including the PSS score. The comparison was performed using the “Anova” method from the “lmerTest” Rpackage. To analyze the fixed effects and interaction effects, the chi-square test statistic (χ^2^_df_) and *P* value were calculated.

## Results

### Population Characteristics

Table 1 shows the population characteristics for all participants included in the analysis. A total of 328 participants fulfilled the criteria of having complete ECG data from Friday 12 AM to Monday 4 PM. There were slightly more male participants (n=186, 56.7%) than female participants (n=142, 43.3%) in the analysis. The Mann-Whitney-Wilcoxon test showed that between male and female participants, there were significant differences in the PSS score (W=1.61e^4^, *P*<.001) and the median HR (W=1.67e^4^, *P*<.001), but not in age (W=1.46e^4^, *P*=.10). The Wilcoxon signed-rank test showed that there was a significant difference in median HR over 24 hours for workdays compared with weekend days (V=2.23e^4^, *P*=.007).

**Table 1:** Descriptive statistics for the population characteristics (N=328).

Characteristics		Median (IQR)	Comparison test result^a^
**Age (years)**			W=1.46e^4^, *P*=.10
	Male (n=186)	37.0 (±16.8)	
	Female (n=142)	39.0 (±15.8)	
	Total (n=328)	38.0 (±17.0)	
**PSS^b^ score**			W=1.61e^4^, *P*<.001
	Male (n=186)	13.0 (±9.0)	
	Female (n=142)	15.0 (±9.0)	
	Total (n=328)	14.0 (±9.0)	
**HR^c^ (bpm^d^)**			
	Male (n=186)	72.5 (±12.0)	W=1.67e^4^, *P*<.001
	Female (n=142)	75.8 (±10.0)	
	Workdays (n=328)	73.6 (±12.3)	V=2.23e^4^, *P*=.007
	Weekend days (n=328)	73.7 (±10.9)	
	Total (n=328)	73.5 (±10.8)	

^a^For comparing male and female participants, the Mann-Whitney-Wilcoxon test was used. For comparing workdays and weekend days, the Wilcoxon signed-rank test was used.

^b^PSS: Perceived Stress Scale.

^c^HR: heart rate.

^d^bpm: beats per minute.

### Final Model

In the final model, 15,699 out of 15,744 observations (99.71%) were included after exclusion of NA values. The predictors included in the final model (AIC=100379.1, LogLik=−50043.6) were (1) the hour of the day, (2) the circadian harmonic, (3) the 12-hour harmonic, (4) the 8-hour harmonic, and (5) the 6-hour harmonic as time components; (6) whether it was the weekend as a within-subject variable; and (7) gender, (8) the PSS score, and (9) age as between-subject variables.

The two-way interaction effects included the following: whether it was the weekend × every harmonic over time; gender × 24-hour harmonic; age × 24-hour harmonic; PSS score × 24-hour harmonic; gender × PSS score; and age × whether it was the weekend.

The following two three-way interaction effects were included in the final model: gender × PSS score × 24-hour harmonic; and age × whether it was the weekend × 24-hour harmonic. The final model, including these predictors, had the lowest AIC value compared with all other models possible with the same main effects. In Multimedia Appendix 2, the built of the final model is shown with AIC and LogLik values for every layer. The final model significantly outperformed the same model without the PSS score and its interactions as predictors (AIC=100381.0, LogLik=−50050.51), according to an Anova model comparison (χ^2^_6_=13.88, *P*=.03).

### Random Intercepts and Slopes

The random intercepts per participant (χ^2^_1_=6948.7, *P*<.001) and for workdays and weekend days (χ^2^_1_=233.9, *P*<.001) both significantly improved the model. Adding random slopes for the time of the day (χ^2^_4_=391.6, *P*<.001); the circadian effect of time (χ^2^_14_=1294.3, *P*<.001); and the harmonic over 12 hours (χ^2^_22_=668.7, *P*<.001), over 8 hours (χ^2^_30_=148.3, *P*<.001), and over 6 hours (χ^2^_38_=361.9, *P*<.001) significantly improved the model of HR. Adding an autoregressive correlation of HR over time also significantly improved the model (χ^2^_1_=12097.4, *P*<.001).

### Main Effects of Time, Weekend, Gender, Age, and PSS Score

There were significant correlations with the HR for the main effects of the hour of the day (χ^2^_1_=73.22, *P*<.001); the circadian harmonic (χ^2^_2_=284.4, *P*<.001); the harmonic over 12 hours (χ^2^_2_=242.1, *P*<.001), over 8 hours (χ^2^_2_=23.78, *P*<.001), and over 6 hours (χ^2^_2_=82.96, *P*<.001); and gender (χ^2^_1_=24.02, *P*<.001). Table 1 shows a significant difference in HR for gender. However, whether it was a workday or weekend day was not significantly associated with HR (χ^2^_1_=1.13, *P*=.29). The age (χ^2^_1_=0.019, *P*=.89) and PSS score (χ^2^_1_=0.42, *P*=.52) of the participant also did not have a significant correlation with HR.

### Two-Way Interaction Effects With Circadian Harmonic Over Time

**Figure 2:**
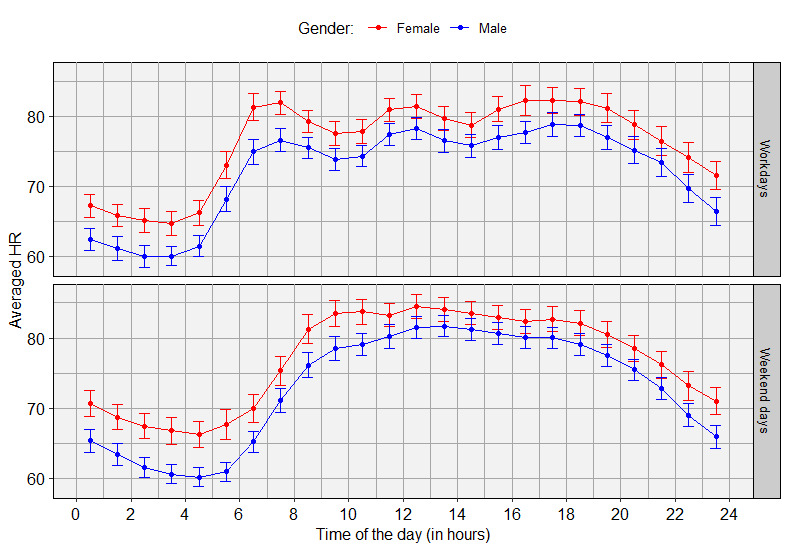
The heart rate (HR) over the time of the day, split for gender and workdays and weekend days. Since the data are summarized per hour, data points are provided in the middle of the hours. The error bars represent the 95% confidence intervals.

There were significant correlations with HR for the interaction effects of weekend and the circadian harmonic (χ^2^_2_=24.98, *P*<.001), weekend and the harmonic over 12 hours (χ^2^_2_=112.5, *P*<.001), weekend and the harmonic over 8 hours (χ^2^_2_=94.07, *P*<.001), and weekend and the harmonic over 6 hours (χ^2^_2_=131.4, *P*<.001). In Figure 2, HR is shown over the time of the day separately for gender and workdays and weekend days.

Furthermore, a significant correlation with HR was found for the interaction effect between gender and the circadian harmonic (χ^2^_2_=17.01, *P*<.001) and between age and the circadian harmonic (χ^2^_2_=16.69, *P*<.001). Although the interaction effect between the PSS score and the circadian harmonic was not significantly correlated with HR (χ^2^_2_=2.62, *P*=.27), it was included in the final model because of a significant higher order interaction effect.

### Two-Way Interaction Effect Between Age and Weekend, and Between Gender and the PSS Score

The correlation between HR and the interaction effect of gender and the PSS score of the participant was slightly nonsignificant (χ^2^_1_=3.63, *P*=.06). It was still included in the model, because of a significant higher order interaction effect. Although there was no significant correlation with HR for the interaction effect between age and whether it was the weekend (χ^2^_1_=0.34, *P*=.56), the interaction effect was included in the final model, because of a higher order interaction effect.

### Three-Way Interaction Effect Among Age, Weekend, and Circadian Harmonic

**Figure 3:**
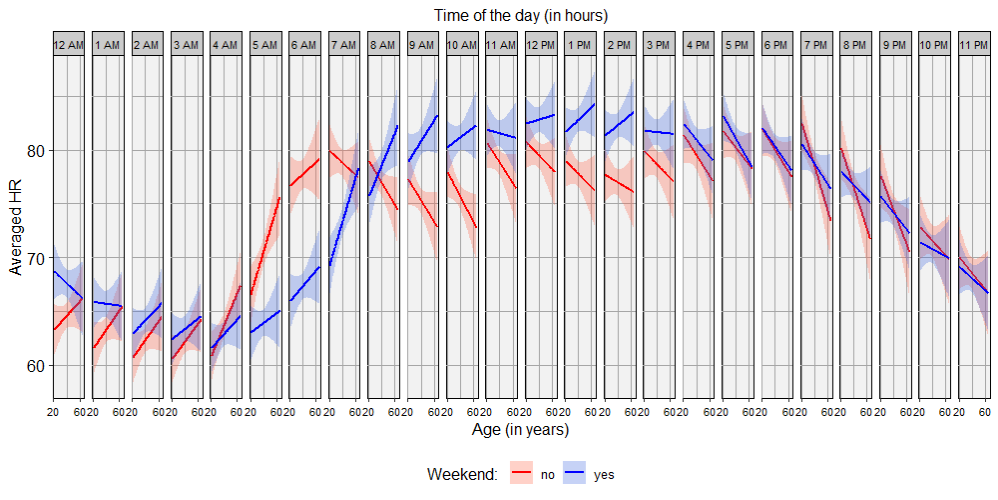
The heart rate (HR) over age per hour of the day, split for workdays and weekend days. Since the data are summarized per hour, the labels provided are the start of the hour.

There was a significant correlation between HR and the three-way interaction effect among age, whether it was a workday or weekend day, and the circadian harmonic (χ^2^_2_=7.13, *P*=.03). Figure 3 shows the effect of age on HR for every hour of the day, split for workdays and weekend days. On workdays, the correlation between age and HR switched from positive to negative at around 7 AM, whereas on weekend days, the same correlation switched from positive to negative at around 3 PM.

### Three-Way Interaction Effect Among Gender, PSS Score, and Circadian Harmonic

**Figure 4:**
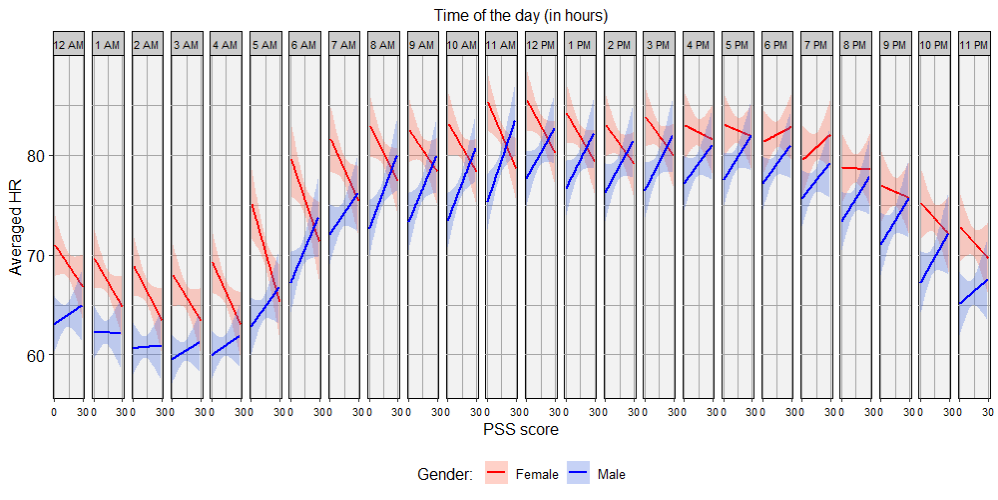
The heart rate (HR) over the Perceived Stress Scale (PSS) score for every hour of the day, split for female and male participants. The time above each subsection provides the starting time of the hour for which the HR is shown.

The three-way interaction among gender, the PSS score, and the circadian harmonic was found to be significantly associated with HR (χ^2^_2_=7.59, *P*=.02). Figure 4 shows the effect of the PSS score on HR for every hour of the day, split for female and male participants. The positive correlation between the PSS score and HR for male participants was the strongest at around 11 AM and flattened in the night. The negative correlation between the PSS score and HR for female participants was the strongest in the night/early morning and flattened or even switched to a slightly positive correlation at around 6 PM to 8 PM.

## Discussion

### Principal Findings

In this observational study, the circadian rhythm was defined as a combination of the 24-hour harmonic, 12-hour harmonic, 8-hour harmonic, and 6-hour harmonic, which were all found to be predictors of HR over time. The median HR was found to be different for male and female participants and for weekdays versus weekends. A relationship between HR and the three-way interaction of age, the circadian harmonic, and whether it was the weekend was found. Moreover, HR was found to be related to the three-way interaction of the PSS score, gender, and the circadian harmonic.

The results confirm the validity of a four-harmonic circadian rhythm in HR as described by Morelli et al [21]. This is shown by the random slopes in harmonics over 24, 12, 8, and 6 hours and by the main effects of the four harmonics. However, four-harmonic circadian HR fluctuations were different between workdays and weekend days, as shown in Figure 2. Moreover, the random intercept for weekends and the difference in HR between workdays and weekend days, as shown in Table 1, suggested a different average HR for workdays and weekend days. The fact that there was no main effect of weekend is likely related to the inclusion of weekend as a random intercept. These findings match the findings of Cavallari et al [31] and suggest that specific HR models for workdays and weekend days are needed. Jones et al [17] related circadian HR fluctuations to circadian cortisol fluctuations. An increase in cortisol gives rise to appetite, resulting in food intake and therefore an increase in energy and HR. After eating, the parasympathetic nervous system is activated, which stimulates the gastrointestinal tract for food processing [32]. This activation is accompanied by a decrease in HR [33]. Figure 2 shows that for workdays, the eating times were very similar for everyone, with breakfast between 6 AM and 8 AM, lunch between 11 AM and 2 PM, and dinner between 5 PM and 8 PM. On weekends, the eating times are likely to be less congruent among participants, resulting in a smoothened average over the day. The study population consisted of office workers. All working people follow a very similar work rhythm (go to work, sit at work, have lunch, sit at work, and go home at roughly similar times), whereas on weekends, there is much more variability among people. It needs to be verified how this difference between workdays and weekend days scales to a different kind of working population, for example, shift workers having different daily patterns. Another clear difference in HR between workdays and weekend days was the timing of the HR morning awakening response. For workdays, HR rises earlier than for weekend days, which could be related to the earlier awakening times on workdays [34].

The average HR as well as the four-harmonic circadian HR fluctuations were different for not only workdays and weekend days but also participants, as indicated by the random intercept and slopes per participant. This suggests a need for personalized circadian HR models, which has been discussed previously by Fijorek et al [35]. This personalization is also suggested based on the higher average HR for female participants, as shown in Table 1, and the difference between male and female participants in circadian fluctuations in HR, as shown in Figure 2. According to Sandstede et al [36], the higher average HR for female participants is related to their average lower cardiac mass. Figure 2 shows that at daytime, the HRs of male and female participants are closer to each other than at night, which was also observed by Fijorek et al [35] and Bonnemeier et al [37]. According to Gregoire et al [38], the lower day-night difference for female individuals is probably related to the lower activity of the sympathetic nervous system in women, especially during the daytime.

Since the maximum HR decreases linearly with age [29], a correlation between age and HR might have been expected as well. However, this effect was not found to be relevant. This could be related to participant inclusion as a random intercept, explaining a part of the variance caused by differences in age. Nevertheless, age interacts with the circadian rhythm in HR differently for workdays and weekend days, as shown in Figure 3. There was a decrease in amplitude of HR fluctuations with age for workdays but not for weekend days. Hood & Shimon [39] have published a review on the aging clock and its circadian rhythms, in which they describe a lower amplitude of both cortisol and waking activity fluctuations, resulting in similar lower amplitudes of circadian HR fluctuations, described as flattening of the sinusoidal curve, as age increases. This explains the positive correlation between age and HR until 7 AM for workdays, which turns negative and then turns positive again around 12 AM, as can be seen in Figure 3. A higher nocturnal nadir of cortisol levels for higher ages was found by Van Cauter et al [40] and Sharma et al [41]. The translation of cortisol levels to long-term HR levels was not made in these studies but is used more often in stress studies [17,42]. On weekends, the positive correlation between age and HR turned negative at around 3 PM and then positive again at around 2 AM. The shift of the switch from negative to positive from 12 AM on workdays to 2 AM on weekend days could be related to later sleeping times during the weekend [43]. In general, it seems that the average HR during work nights (12 AM to 4 AM) is slightly lower than that during weekend nights, because people go to sleep later during the weekend. However, this does not explain why the switch from a positive correlation of age and HR to a negative correlation shifted from 7 AM on workdays to 3 PM on weekend days. Between 7 AM and 3 PM, there might be the most variance in activity among participants, since these are free hours during weekend days and work hours during workdays. Activity trackers could provide more understanding on the different directions of the correlation between age and HR for workdays and weekend days.

Lastly, no correlations between HR and the main effect of the PSS or HR and the two-way interaction effects of the PSS and other predictors were found. However, a correlation was found between HR and the three-way interaction of gender, the PSS score, and the circadian harmonic. This suggests that the effect of chronic stress can only be captured by including the dynamics of HR over time. Figure 4 shows that the correlation of the PSS and HR was exactly opposite for male and female participants throughout the day, except for the periods around 1 AM to 3 AM and 6 PM to 8 PM. In the first period, a flattening of the positive correlation between the PSS score and HR was noted for male participants, while in the second period, the negative correlation between the PSS score and HR for female participants switched to a slightly positive correlation. Table 1 shows that beside the difference in HR between male and female participants, there was a difference in the PSS score, which was found by Remor [6]. However, this does not explain the more complex three-way interaction effect including circadian fluctuations. Jones et al [17] described similar results, as seen in Figure 4, only for acute stress responses and explained it as a gender difference in evolution-based energy utilization strategies. Male individuals have a well-studied stress response, often called the fight-or-flight response [44,45]. However, for female individuals, Taylor et al [46] introduced a different response, called the tend-or-befriend response. For long-term stress, the fight-or-flight response means stacking up resources to increase energy that might be needed for an actual fight-or-flight situation. In terms of biology, this means an increase in cortisol, accompanied by an increase in appetite, which results in an increase in food intake and energy, resulting in an increased HR [17]. This male-specific chronic stress response could be related to the correlation of job strain and obesity in male individuals but not female individuals, which was found by Brunner et al [47]. The tend-or-befriend response in female individuals would stimulate nurturing and caring behaviors, making sure that, for example, their children have enough resources [46]. This is related to a decrease in food intake, causing the depletion of fat stores and a decrease in HR among female individuals [17]. In the literature, this female evolutionary behavior has also been described as the biological reason why female individuals have, in general, higher fat storage than male individuals [48]. The theory explains the positive correlation between the PSS and HR for male participants and the negative correlation between the PSS and HR for female participants. In Figure 4, the period from 6 PM to 8 PM, when the correlation between the PSS and HR switched from negative to positive for female participants, could be explained by dinner time, which includes food intake among female individuals as well. The period from 1 AM to 3 AM, when the positive correlation between the PSS score and HR for male participants flattened, can be explained by the absence of food intake at night and no increased HR associated with chronic stress during that period. This theory should be studied in more detail in relation to circadian HR, comparing the circadian physiology of male and female individuals for different stress levels and linking HR to cortisol, sleep, and mealtime as well.

### Limitations

This study only involved two workdays (Monday and Friday) and two weekend days (Saturday and Sunday) per participant. For the workdays, Monday stopped at 4 PM. The effect of this limitation can easily be seen in Figure 4, where the CIs in the plots seem to increase from 4 PM. It would be interesting to include more workdays and weekend days of multiple weeks, so an average over multiple days could provide a more consistent long-term measurement of HR fluctuations.

### Future Work

The autonomic stress response includes several physiological changes, of which we investigated the HR response. Our longitudinal analysis should be applied to other physiological signals, such as skin temperature, galvanic skin response, and blood pressure [5,13,14]. These physiological measurements are known to have circadian fluctuations as well. It is also recommended to include longitudinal cortisol measurements to better understand the interaction across individual characteristics, hormonal mechanisms, and physiology in chronic stress. Information on sport activities, food intake times, and sleep times could also be considered to provide more context regarding physiological changes.

### Conclusions

This study confirmed previous findings on the circadian rhythm of HR, its difference between workdays and weekend days, and its interactions with gender and age. The main discovery is the relationship between HR and the three-way interaction of chronic stress, gender, and the circadian harmonic. Our findings suggest that chronic stress prediction models and objective chronic stress measurements based on continuous HR detection should include interaction effects with circadian harmonics and gender to explain more subject variability. The development of these prediction models would enable continuous monitoring of long-term stress levels that could support therapists and psychologists to better understand patient progress and well-being.

## Appendix

Multimedia Appendix 1 Formulas.

Multimedia Appendix 2 Models.

## Abbreviations

ACC: accelerometer
AIC: Akaike information criterion
ECG: electrocardiogram
HR: heart rate
LogLik: log likelihood
NA: not applicable
PSS: Perceived Stress Scale
SWEET: Stress in Work Environment
