# Supplementary material for: Relationship Between Chronic Stress and Heart Rate Over Time Modulated by Gender in a Cohort of Office Workers: Cross-Sectional Study Using Wearable Technologies
Source: J Med Internet Res. 2020 Sep 9;22(9):e18253. doi: 10.2196/18253 (PMC7511872; doi:10.2196/18253)
Supplement: Multimedia Appendix 1 [file jmir_v22i9e18253_app1.docx]

## **Supplementary Materials - Formulas**

The formulas to calculate the features and filters based on the patch data.

The calculation of HR:

$$HR=\frac{number of R.peaks}{window length in minutes}$$

The calculation of MXYZ, n is the total number of datapoints within the time window:

$$MXYZ= \frac{\sum_{i=1}^{n} \left( x_{i}*y_{i}*z_{i} \right)}{n}$$

The calculation of AVGM:

$$AVGM= \frac{\sum_{i=1}^{n} \left( \frac{x_{i}+y_{i}+z_{i}}{3} \right)}{n}$$

(n is the total number of datapoints within the time window.)

The calculation of AVGSD:

$$AVGSD= \sqrt{\frac{\sum_{i=1}^{n} \frac{\left( \left( x_{i}-\bar{x} \right)^{2}+\left( y_{i}-\bar{y} \right)^{2}+\left( z_{i}-\bar{z} \right)^{2} \right)}{3}}{n-1}}$$

(n is the total number of datapoints within the time window.)

Assessment of the maximum HR per individual according to the formula of Tanaka et al.:

$$maximum HR=208-0.7*age$$

Tanaka H, Monahan KD, Seals DR. Age-predicted maximal heart rate revisited. Journal of the American college of cardiology 2001;37.1:153-156. DOI: <https://doi.org/10.1016/s0735-1097(00)01054-8>
