# Supplementary material for: Relationship Between Chronic Stress and Heart Rate Over Time Modulated by Gender in a Cohort of Office Workers: Cross-Sectional Study Using Wearable Technologies
Source: J Med Internet Res. 2020 Sep 9;22(9):e18253. doi: 10.2196/18253 (PMC7511872; doi:10.2196/18253)
Supplement: Multimedia Appendix 2 [file jmir_v22i9e18253_app2.docx]

## **Supplementary Materials - Models**

Table Supplementary Materials - Models. All models examined in built-up of final model, including AIC (Akaike Information Criterion) and LogLik (log likelihood) as criteria.

| Model name | Model description | AIC | LogLik |
| --- | --- | --- | --- |
| interceptM | *no predictors no random intercepts or slopes no autoregressive correlation* | 125584.2 | -62790.12 |
| baselineM | *+ random intercept per participant* | 118637.6 | -59315.79 |
| rand_intM | *+ random intercept per weekend (yes/no)* | 118405.7 | -59198.84 |
| TimeM | *+ hour of the day* | 116136.1 | -58063.06 |
| rand_slope_TimeM | *+ random slope for hour of the day* | 115752.6 | -57867.29 |
| auto_regressiveM | *+ autoregressive correlation matrix* | 103657.1 | -51818.56 |
| rand_slope_Time.H1M | *+ random slope for 24h-harmonic* | 102390.8 | -51171.41 |
| rand_slope_Time.H2M | *+ random slope for 12h-harmonic* | 101766.1 | -50837.05 |
| rand_slope_Time.H3M | *+ random slope for 8h-harmonic* | 101677.8 | -50762.90 |
| rand_slope_Time.H4M | *+ random slope for 6h-harmonic* | 101391.9 | -50581.93 |
| Time_H1M | *+ 24h-harmonic* | 101111.4 | -50439.72 |
| Time_H2M | *+ 12h-harmonic* | 100873.4 | -50318.67 |
| Time_H3M | *+ 8h-harmonic* | 100853.6 | -50306.78 |
| Time_H4M | *+ 6h-harmonic* | 100774.6 | -50265.30 |
| weekendM | *+ weekend (yes/no)* | 100775.5 | -50264.74 |
| genderM | *+ gender* | 100753.5 | -50252.73 |
| ageM | *+ age* | 100755.5 | -50252.74 |
| PSSM | *+ PSS* | 100757.1 | -50252.53 |
| weekend.H1M | *+ weekend (yes/no) × 24h-harmonic* | 100736.1 | -50240.03 |
| weekend.H2M | *+ weekend (yes/no) × 12h-harmonic* | 100627.6 | -50183.80 |
| weekend.H3M | *+ weekend (yes/no) × 8h-harmonic* | 100537.5 | -50136.76 |
| weekend.H4M | *+ weekend (yes/no) × 6h-harmonic* | 100410.1 | -50071.08 |
| gender.H1M | *+ gender × 24h-harmonic* | 100397.1 | -50062.57 |
| age.H1M | *+ age × 24h-harmonic* | 100384.4 | -50054.23 |
| PSS.H1M | *+ PSS × 24h-harmonic* | 100385.8 | -50052.92 |
| gender.PSSM | *+ gender × PSS* | 100384.2 | -50051.10 |
| age.weekendM | *+ age × weekend (yes/no)* | 100385.9 | -50050.93 |
| gender.PSS.H1M | *+ gender × PSS × 24h-harmonic* | 100382.3 | -50047.14 |
| age.weekend.H1M | *+ age × weekend (yes/no) × 24h-harmonic* | 100379.1 | -50043.58 |
